# Patient involvement in a qualitative meta-synthesis: lessons learnt

**DOI:** 10.1186/s40900-016-0032-0

**Published:** 2016-05-12

**Authors:** Kerin Bayliss, Bella Starling, Karim Raza, Eva C. Johansson, Codruta Zabalan, Susan Moore, Diana Skingle, Tiina Jasinski, Susan Thomas, Rebecca Stack

**Affiliations:** 1 Public Programmes, Central Manchester University Hospitals NHS Foundation Trust, University of Manchester, Manchester Academic Health Science Centre, Manchester, UK; 2 Centre for Translational Inflammation Research, College of Medical and Dental Sciences, University of Birmingham, Birmingham, UK; 3 EuroTEAM Patient Research Partner, Birmingham, UK; 4 Rheumatoid Arthritis service user group, University of Manchester, Manchester, UK; 5 Division of Psychology, School of Social Sciences, Nottingham Trent University, Nottingham, UK

**Keywords:** Patient and public involvement, Qualitative research, Meta-synthesis, Review, Evaluation, Co-researcher, Co-production

## Abstract

**Plain English summary:**

Patients and researchers must work together to improve the relevance and quality of research. Qualitative systematic reviews synthesise findings from a range of published qualitative studies to identify common themes, and can make recommendations for practice or future research. The process of conducting a systemic review offers multiple opportunities for patient involvement.This paper explores the reflections of patient research partners involved in a qualitative systematic review. Patient partners were asked how their experience of the review process could be used to improve patient involvement in future qualitative systematic reviews. Following involvement in a systematic review an exploratory questionnaire was emailed to eight patient research partners. Open ended questions focussed on the training they had received, whether they had enjoyed taking part and how the process could be improved. Patients stated that they needed clear instructions and examples of how to take part in the systematic review. Face to face training was preferred, and it was important that patients were given enough time to complete the task. The study led to benefits for patients including gaining new skills and improved confidence. Each patient also wanted to know how their comments had influenced the paper and wanted feedback on whether they needed any further training. Through reflection with patient partners, recommendations for the involvement of patients in qualitative systematic reviews were developed to allow researchers to successfully involve patients in the review process.

**Abstract:**

**Background**

Patient involvement in systematic reviews is seen as good practice, yet there is a lack of accessible standardised training for those involved. The aim of this paper is to inform the evidence base on effective ways of involving patients in a qualitative meta-synthesis. This process is evaluated and reflected by patient research partners (PRPs) who provided accounts of their experience.

**Methods**

An open ended questionnaire was emailed to eight PRPs who had participated in the analysis of a qualitative meta-synthesis. Questions focussed on the training they received, their experience of coding data and identifying themes, whether they enjoyed taking part in the project and how the process could be improved.

**Results**

Our findings point to the importance of detailed training for PRPs, using plain English and clear examples of analysis techniques to improve confidence in engaging with meta-synthesis methods. Face to face training was preferred in order to discuss a PRP’s understanding of the task ahead. Time is an important consideration as PRPs often complete this work on top of their daily commitments and need the time and on-going support to be able to immerse themselves in the data. A focus group was a useful way to discuss the themes but it is important that PRPs understand how their comments have influenced the paper. PRPs reported benefits that included building new skills, improving confidence and gaining knowledge. They also asked for feedback on their contribution and any further training needs. All PRPs said they would take part in a meta-synthesis in the future as long as these considerations were addressed.

**Conclusion**

The recommendations for practice identified in this paper, and guidelines for training, can assist researchers in collaborating with PRPs when developing and conducting a qualitative meta-synthesis.

## Background

The concept of patient and public involvement (PPI) in research is now well-accepted as a way to enhance the relevance, validity and quality of research [1]. PPI roles often include patients drawing on their personal experience to suggest ways that could improve a project or service. However, some PPI roles, such as involvement in a systematic review, can also require specialist knowledge about the research process. This therefore goes beyond personal experience and asks the patient to invest time in training to develop their knowledge, skills and experience to facilitate effective input [2–7].

INVOLVE [2], which is the UK-based advisory body on PPI in health and social care research, encourages researchers to involve patients in systematic reviews by highlighting the benefits of this activity. For example, INVOLVE states that patients who know about the topic of the review and have first-hand experience of the disease and treatment can be involved in setting the scope of the review in terms of what is a priority to them; providing a different perspective on how to understand an idea; interpreting findings; and improving ‘readability’ of scientific language and jargon [3]. Patients can be involved to different degrees from a consultation which includes a one off meeting to discuss the focus or content of a review [8], to a collaboration where patients are involved in the research design, data analysis and interpretation [9, 10]. However, this level of partnership is rare [9, 10].

Dudley and colleagues [11] report that very few patients are involved in systematic reviews and the evidence for how best to do it is still lacking. Most PPI training courses are developed within particular organisations or in the context of individual research projects [2], and there have been few empirical studies of research training needs for PPI contributors [11]. This can mean that researchers often reinvent the wheel when involving patients in systematic reviews due to a lack of accessible standardised training [11, 12].

This paper aims to evaluate the patient experience of involvement in a qualitative meta-synthesis. This is important as the promotion of PPI activity in research, rather than its evaluation, has been the focus of much of the published literature [6]. Previous projects have highlighted the value of listening to the accounts of PPI contributors in order to highlight good practice and areas in need of improvement [13]. Furthermore, by evaluating the training developed for this study, the authors will help to ensure its relevance and therefore encourage uptake and a more consistent approach to PPI in systematic reviews.

## Methods

### Context

In 2014, the authors collaborated with eight patient research partners (PRPs) to complete a meta-synthesis of qualitative studies to explore perceptions of predictive testing for those at risk of developing a chronic inflammatory disease [14]. This study was part of the EuroTEAM project [15] and used meta-synthesis methods to investigate common themes and implications for future predictive testing programmes. The PRPs involved in this study were from a number of European countries (UK, Sweden, Estonia and Romania), and included PARE (People with Arthritis/Rheumatism in Europe) board members and founders and members of national rheumatic and RA patient organisations. The PRPs were initially recruited to give academic and industry partners feedback on research and provide an insight into the experiences of Rheumatoid Arthritis patients. They attended a PRP launch event as part of the wider EuroTEAM study, where they were made aware of what their role as a PRP would entail, and the nature of the research they were taking part in. This group were highly articulate, all female, and had previous experience of being a PRP as they had been involved in the larger EuroTEAM study, and contributed towards other research activities within that study. This meant that they had an understanding of the research process. An additional PRP was recruited from a Rheumatoid Arthritis service user group at the University of Manchester.

All PRPs were invited by email to take part in the meta-synthesis. The researchers did not collaborate with PRPs when developing the research question, although the PRPs were involved in the writing of the EuroTEAM grant application. PRPs also had some input into the search strategy as the researchers provided updates on the progress of the review via teleconference calls while the searches were completed and the papers selected. This helped PRPs to gain familiarity with the review process. A second email asked for three volunteers to help to develop a coding framework for the data in order to co-produce the themes with the researchers. Three qualitative papers were selected at random from the 11 papers included in the review and were sent to the first three volunteers.

In line with recommendations [2], we developed training that was tailored to the roles of patients, in the form of a set of written instructions on how to code the results sections (Appendix 1). It was necessary to develop written materials rather than face to face training as the PRPs were based across Europe. The training material was developed from the qualitative methods teaching material delivered to undergraduate students and trainee doctors with no qualitative experience. This included examples of qualitative research and meta-syntheses techniques. The material was shortened, and graphic representations were added. Although research experience varied within the group, the authors decided to prepare the materials with the view that all PRPs were new to qualitative research. This promoted consistency in the techniques that were used. The first draft of the materials was shown to a PRP, who highlighted any sections that were unclear or ambiguous. The authors then made the necessary changes before circulating. All PRPs were given three weeks to complete this task and were also given the option within the email of a phone call to talk through the guidance if required. Their feedback and recommendations supported the researchers’ coding framework and no substantial changes were made.

The coding framework was used by the researchers to complete a thematic analysis of the data. A third email was sent to the PRPs to ask them to review these themes. A training document was attached on how to identify themes (Appendix 2) and they were given two weeks to read the 11 papers and complete the task with the option of phone support. The email also invited the PRPs to attend a 90 min face to face focus session at the EuroTEAM annual UK meeting on 6th February 2014. This session aimed to help researchers draw on the perspectives of PRPs when interpreting and reflecting upon the data [16]. Researchers presented the preliminary themes and corresponding quotes to the PRPs. The following questions were then discussed in order to facilitate active engagement and reflection in the development of themes:

1. Do these themes and subthemes look logical? (For example, do the themes make sense to you as a person who has experience of a chronic inflammatory condition. Does the information flow?).
2. Based on your experience as a patient or relative do these themes reflect reality? (For example, do you think we have missed any themes in our analysis?).
3. Do you think there are any important concepts described within these subthemes?
4. Do you think that the overall themes or subthemes can be changed in anyway?
5. Do you think that our meaning/interpretation of the quotes within the papers can be changed?
6. What could be the implications of these findings for those at risk of developing a chronic illness in the future?

The aim of the session was to get patient feedback on whether they thought that the researchers had missed any important themes, or highlight whether they would have interpreted the data in a different way. The answers to the questions supported the themes presented by the researchers and highlighted the need to report clear recommendations for practice. PRPs helped to prioritise the data by choosing which quotes were included in the paper, organise themes and develop recommendation for practice, discussing the practicalities of how a new or improved intervention programme would work. Patients brought their own experience and helped the recommendations come to life, ensuring they were acceptable and valuable to patients. Considerations such as the emotional impact of any interventions were also discussed [14].

### Evaluation

An open ended questionnaire (available from the authors) was developed to collect data about the PRP’s experience of collaborating with researchers to complete a qualitative meta-synthesis. Questions were developed from a review of the literature and discussions within the research team. The eight PRPs who worked on the meta-synthesis were sent this questionnaire by the researchers via email. Questions focused on the patients’ experience of training for this activity, what they gained from the experience and how the process could be improved. The email, which was sent in June 2015, also contained information on the process of the review to refresh their memory: what they had been asked to do and when, and the instructions they had received. All patients were given four weeks to complete the evaluation.

Analysis was inductive, using thematic analysis [17]. Disconfirming evidence was actively sought and was used to modify emerging themes. The questionnaires were read, annotated and categorised independently by KB and RS. The researchers then met to discuss the themes. To support our interpretations, we present illustrative extracts from the data.

## Results

Feedback was received from six out of the eight PRPs involved in this research. Six themes were identified from the questionnaire data: understanding the research process; the need for face to face training; time provided for each task; the focus group to discuss themes; providing feedback and personal benefits for the patients.

### Understanding the research process

Patients provided positive feedback on the instructions for both the coding and thematic analysis activities (Appendix 1 and 2), stating that they were clear and helpful. These documents included text and images to explain how to code data and identify themes:

The document is well laid out, is easy on the eye and encourages fluent reading. I particularly like the recommendations and considerations advice. The tips I suppose helps to prevent you from ‘wandering off’, the point. I find that both examples on how to summarise are explained very well, are simple and easy to follow (Patient research partner 3).

…the two different style examples of coding were very good (Patient research partner 1).

Yes. It was clearly laid out (Patient research partner 5).

When coding the data, all patient research partners in this study felt confident completing the task. A phone call was offered to go through the materials, and although patients valued this offer, only one person chose to take this up:

I remember we were sent the explanatory materials and offered a skype or phone call talk with the researcher/the specialist, which was very helpful in letting me know what the researcher expects from me (Patient research partner 1).

However, one patient research partner stated that some of the language used in the guidance materials was not clear. It was believed there was a need to explain some of the words associated with a qualitative meta-synthesis in a manner more understandable to a lay audience. Furthermore, while the patients all had rheumatoid arthritis, the meta-synthesis also included papers which focused on other chronic inflammatory conditions. Patients stated that they needed more support in understanding the terminology in the papers on these other conditions, especially when English was not their primary language. Gaining some background information on the previous experience of each patient to gauge the level of support they may need would have been beneficial:

Yes, some simplified explanations of the relevance and use of ‘themes’, ‘sub-themes’ and ‘coding’ would have been useful. An understanding of “a qualitative literature synthesis” is not a given (Patient research partner 4).

It was very difficult to comprehend fully research documents dealing with conditions other than RMDs [Rheumatic and Musculoskeletal Diseases] without an appropriate glossary etc. (Patient research partner 4).

The training consisted of reading the instructions ‘Patient involvement in the initial coding of data from selected qualitative papers’ which I found instructive and well written, but one thing confused me from the start and still do: the terminology. Coding is used in the title and summarise in the body text, but what I actually did I think of as writing comments on the text. English isn’t my native tongue so maybe I get more easily confused. I would like a short run through of the differences between these terms (to code, summarise, comment) and also an explanation to things like *n* = 9 that you immediately meet as you start reading the papers themselves (Patient research partner 2).

Other patient research partners drew from their own experiences to help them make sense of the task. However, they remained unsure of exactly what was required of them. A clearer example of feedback from a paper that had already been coded was suggested as it was thought that this would reduce subjectivity and clarify the process.

…I felt like I was doing an ordinary text analysis just as I do when translating drama, when you look for what is being said and what is really being said. I felt very unsure of whether that was what you wanted. Would have needed feedback (Patient research partner 2).

And I would have liked to receive on email (as a training material) an example of a paper coded as to answer exactly the researcher’s needs (Patient research partner 1).

### The need for face to face training

Patient research partners stated that face to face training would have been more beneficial than the written instructions provided as it would have allowed them to ask questions and learn from others. This could have been achieved by a video conference or skype call with multiple participants. Another alternative could have been a live online training module where the researcher presented how to complete the task and patients could type their questions during the session. Although patients were instructed to focus on the results section of the papers, there was still some confusion on which part of the article was most relevant to the task:

Maybe a talk with the researcher and the other patient partners (so that we can raise questions). Also some training on how to read an article (for example, is it better to focus more on the Abstract, Discussion and Conclusion or is it better to read carefully the whole document in order not to miss important issues/ideas?) (Patient research partner 1).

Perhaps face to face (training) to explain the process and to answer questions [would be better] (Patient research partner 3).

Feedback was provided in varying detail from the PRPs. Further guidance from the researcher could have elicited more uniform responses:

I would have liked to go together with the researcher (face to face) through one page or so from the materials to be coded so that I am confident I’m doing the right thing (Patient research partner1).

### Time provided for each task

When asked how long it took to code three papers for the first task (Appendix 1), patient research partners reported that it took between three and seven hours. These three individuals felt that they were given enough time to complete this task (three weeks), and were able to work around their existing commitments:

I had to read it a number of times and come back to it quite often so about 3 hour’s ish (Patient research partner 3).

I remember that I didn’t do all the work at one sitting; it depended on the spare time I had (because of my part-time job and family commitments) (Patient research partner 1).

However, PRPs involved in this study stated that they would have liked more time to complete the second task (Appendix 2). Due to tight deadlines, patients were given two weeks to read the papers in preparation for the focus group. Only one individual believed that they had enough time to complete this activity. The other patients believed that this was not enough time for them to work through the papers when they had other family and work commitments. One respondent suggested that two months would be a more realistic timeframe:

Instructions came very late together with the 11 papers. An immense amount of papers with too short a deadline. I’m sorry but I had no time to read or prepare before the meeting. To be able to do this well together with our ordinary jobs at least two months (11 papers!). Thinking needs that time I think (Patient research partner 2).

Sending 11 documents for reading before a meeting the following week was unrealistic, for me at least. In fact there was so much data that my inbox would not accept it (Patient research partner 4).

No, I didn’t [have enough time]. I remember I was working the whole duration of the flight. Taking into consideration that there were many articles, I would have liked to have two-three weeks at my disposal (this is also because of my part-time job and the fact that sometimes I get to work only at weekends) (Patient research partner 1).

### The focus group to discuss themes

For those patient partners who had read the papers, they found that the focus group was useful and a good way to discuss the themes. They believed that the data was clearly presented and they had an opportunity to contribute:

Yes, indeed. The researcher conducting the group was very attentive in offering everybody the opportunity to speak (Patient research partner 1).

Yes [I had an opportunity to share my views]. Relaxed group (Patient research partner 5).

Potential improvements included writing comments down as the session progressed so that patients could see how they were contributing to the analysis:

Maybe working with a white board more and writing down patients’ ideas so that have a holistic view… A synthesis of the group discussion so that patients see if and what they were able to contribute to the draft paper, would have boosted patients’ confidence in their input and knowledge/value (Patient research partner 1).

When patients did not have time to prepare for the activity they felt that the focus group to discuss the themes was a frustrating exercise. Patients suggested that they could have sent their ideas to the researcher before the focus group so this data could be collated and presented for discussion on the day:

I found this a rather confusing… I had no idea what coding was, how the themes had been chosen, nor how they might be used, or really what we were trying to achieve. I think the whole process was a bit opaque (Patient research partner 4).

If the articles had been sent [further] in advance, the researcher could have summarized everybody’s view/ideas in the “Draft Themes and Sub Themes” and when gathering together as a focus group we could have only rated and improved the document (Patient research partner 1).

### Providing feedback

The researcher thanked the PRPs for their work but PRPs highlighted that they would have liked more detailed feedback including whether any improvements could be made. This highlights the need for researchers to regularly consult with PRPs throughout the project to ensure that their needs are being met rather than waiting for patients to ask for help:

A quick feedback after paper 1 to make sure I was doing what you wanted, with further requests from the researchers if needed. We PRP’s want to help the best we can (Patient research partner 2).

I would have liked some quick feedback… to get a notion of whether I could improve anything, whether I had understood the instructions (Patient research partner 2).

I would have liked to have another call with the researcher [to] explain to me what was good and what was wrong and give me examples/ideas on how to improve what was wrong (Patient research partner 1).

The evaluation for this project was conducted 15 months after the event due to the researcher going on maternity leave. Patient research partners stated that this gap meant that they sometimes struggled to remember the details of taking part in the study:

For the future it would be very helpful if we can fill in the evaluation forms in one or two months after completing the activity (Patient research partner 1).

### Personal benefits for patient research partners

The PRPs in this study reported that their involvement had personal benefits which included building new skills, improving confidence and gaining knowledge.

Taking into consideration that it was the first activity of that kind in my experience as a Patient Research Partner, I was very happy and thankful to gain experience in coding. I improved my skills in summarizing, finding key words and interpreting. I gained confidence in participating to future coding activities. It was also very interesting to see how other people think about RA and prevention – very educative (Patient research partner 1).

I feel I am contributing and being nosey; I get to find things out (Patient research partner 3).

[Benefits included the] sharing of ideas and experiences (Patient research partner 6).

All patients said that they would like to take part in both activities again if they were given adequate time and support:

Certainly, yes! [I would take part again] I liked it (Patient research partner 1).

However, one patient questioned the real benefit of their contribution to a qualitative meta-synthesis. This highlights a need to further explain the reason for the patient’s involvement and the value of their input, and maybe further training to ensure patients feel that they have the skills to make a positive contribution:

To be honest, I’m not quite sure. I don’t know… if I was useful really, not in a general way but more specific. I think the idea of involving patients in this kind of work is potentially very good, but I had a constant lurking feeling of just being an amateur scientist (Patient Research partner 2).

## Discussion

This study provides an evidence base on effective ways to involve PRPs in a qualitative meta-synthesis. This follows the recommendations of previous research which highlights a need for quality standards and recommendations for good practice in the involvement of patients and carers in this area [18–21]. The importance of this work is also highlighted by Harris et al. [19] who states that a lack of involvement in systematic reviews may compromise the validity of the reviews as researchers may not always identify all of the important themes.

Patient research partners were asked to evaluate their experience of completing training to collaborate with researchers to develop a coding framework; complete a thematic analysis of qualitative data; and advise on the layout and focus of a paper. We have used the insights of PRPs to generate the recommendations below which may help researchers to involve patients in future systematic reviews. These recommendations should be considered alongside existing guidance on PPI [2], and may also assist funding bodies when reviewing whether plans for PPI in a qualitative meta-synthesis are feasible.

### Lessons learnt: recommendations for PPI in a qualitative meta-synthesis

Please note that all recommendations marked with a ^a^ are generic recommendations to all PPI:

1. ^a^Ensure that all training materials use plain English and include an appropriate glossary.
2. ^a^Ensure that you add adequate time and resources to your project for meaningful PPI.
3. ^a^Identify individual support and training needs before the project starts. For example, the group may contain people with varying understanding of the research process or individuals with English as their second language. Providing clear instructions on what is expected from each patient research partner (PRP) avoids confusion and the feeling that they are acting as a ‘pseudo scientist’.
4. Always ask PRP’s how much time they have to give to the project. For example, if your literature search finds a large number of relevant papers, does each individual have the time to read these? Negotiate the level of involvement that is suitable for your PRPs and the specific project.
5. When asking PRPs to complete a thematic analysis, provide clear examples of the feedback that is required. This can include the level of detail and format of their response. An analysis template can be used to avoid PRPs spending unnecessary time on a task and improve the consistency of feedback so it is easier to collate these data.
6. ^a^A phone call to each PRP after they have been sent any written instructions would be beneficial. Be proactive and do not wait for the patient to contact you with any questions.
7. ^a^Face to face was seen as the preferred method of training in this study. If it is not possible to get all PRPs together then a video conference or live online training session are good alternatives.
8. ^a^Assessed questions at the end of the training session can highlight whether PRPs have understood the training information and ensure that they have an understanding of the task ahead. Further training may be required on areas where they remain unsure.
9. Ask the PRPs what they believe a suitable time frame would be to complete each task considering existing commitments. The PRPs in the current study believe that three weeks was sufficient to code three papers, and two months should be given to analyse 11 papers.
10. It is beneficial to provide the themes identified by the researcher and the questions that patients will be asked before the focus group. This provides a template for patients to work from when completing their own analysis and preparing for the meeting. Patient research partners can then state whether they agree with the themes identified, if anything is missing, how to structure the results section of the paper and highlight priorities.
11. A focus group was useful and a good way to discuss the findings of the thematic analysis. Eight PRPs was a good number to allow a meaningful discussion around the themes identified.
12. Write up all feedback on a flip chart or white board during a focus session and circulate comments.
13. ^a^Thank PRPs for their involvement and highlight the value of their input. State how their contributions will affect the paper.
14. ^a^Ask patients whether they would like feedback on their contribution, including any further training needs.
15. ^a^Evaluate the project at each stage. This helps to identify support needs as the project progresses rather than waiting until the end.
16. ^a^Evaluate the project as a whole as soon as possible following completion so the PRPs are able to clearly recall their experiences.

### Comparisons with existing literature

This study developed a set of training materials for PRPs (Appendices 1 and 2) with the aim of supporting a collaboration between PRPs and researchers in a qualitative meta-synthesis. An evaluation of these materials found them to be clear and helpful, however, PRPs also highlighted that some amendments should be made to these documents if they are to be used as a template for future projects. For example, a glossary should be added to clearly explain all terminology, and a small assessment and evaluation at the end of the training could also identify any further training needs. An analysis template with a clear example of the level of feedback required would also be beneficial and should be developed with PRPs to add to the training. Furthermore, PRPs were recruited from across Europe therefore geographical distance and time constraints meant that training was done remotely by email. However, PRPs stated that face to face training would have been preferred. Buck et al. [13] found that face to face meetings were more conductive to forming relationships with PPI contributors than teleconferences. Therefore a video conference may be a preferred option when PRPs are in different countries. These recommendations are in line with Noe et al. [22] who state that a key consideration for any training is that it engages with the diversity of learners’ needs and is meaningful from their perspective.

Not all PRPs in this study were able to develop an understanding of the research methods due to the existing commitments and limited one to one support. These individuals therefore relied more on their personal experience to make comments and suggestions on the paper rather than analysing the data. This level of involvement is reflected in a systematic review completed by Rees and Oliver [23] where researchers produced a systematic map of the literature and a group of PRPs decided what to focus on and which outcomes should be prioritised. Although PRPs were able to use their personal experience to comment on the paper, they may have had greater input if they had had more time to develop meta-synthesis techniques and analyse the data.

As conducting a review can be very time consuming, PRPs may prefer to develop the focus of the review rather than being involved in the analysis of the data. It is therefore important to negotiate the level of involvement with PRPs before the study begins to ensure that involvement is meaningful and not simply tokenistic [6, 11, 16]. This is supported by Harris et al. [19] who found that although sustained and equal involvement of PRPs in the review process substantially improved the quality of the review, the level of participation should be flexible considering issues of time and cost, researcher skills and experience in PPI; and the circumstances in which the review is funded. This issue points to a ‘know-do’ gap, whereby researchers’ talk of the importance and value of PPI in the ‘ideal’ world in contrast to their experiences of ‘the reality’ of implementing PPI in practice where time restrictions can form barriers to PPI [24]. By considering what is achievable in a given time frame the PRPs and researchers can work together to consider where PRP input is most valuable for individual projects [11, 13].

The authors had very little disagreement with PRPs on the analysis of data during this meta-synthesis. However, if more time had been available for the PRPs to complete the analysis before the focus session, more significant changes to the themes may have been suggested. Further guidance should be developed on what to do when PRPs and researchers have contrasting views about data. For example, Robinson et al. [25] found that it can be difficult for researchers to relinquish control and work with patients to develop themes. Training may be beneficial for researchers on how to meaningfully involve patients in a meta-synthesis/review and manage this distribution of power. Further research is therefore necessary with PRPs to develop training allowing contributors and researchers to learn from each other [2]. Consideration might also be given to training PPI contributors and researchers side-by-side [11]. Buck and colleagues [13] also state that PPI in systematic reviews could also be extended to involve patients in the distribution of research findings to relevant groups.

### Strengths and limitations

To our knowledge, this is the first study to evaluate PRP training and involvement in a qualitative meta-synthesis. The insights of PRPs can be used to help researchers plan PPI in a qualitative meta-synthesis. This follows the recommendations of a recent study of the UK health and social care research community which informed the development of a Public Involvement Impact Assessment Framework (PiiAF). This emphasises the value of well thought-through planning before implementing PPI as well as the subsequent evaluation of its impact [26].

Previous research has found that it can be difficult to find and engage patients with an interest in and understanding of the research [13]. However, the researchers in this study had access to articulate patients, who were already fully engaged in the topic as they had previously worked on other RA projects. This meant that they had an understanding of the research process and the constraints on researchers [11]. Enany et al. [27] state that PPI contributors with higher levels of education may be prone to over identify with the perspectives of researchers rather than challenging them [27]. However, patients do need certain skills to be able to contribute to systematic reviews which include the need to be able to articulate ideas and listen to other people’s ideas and work in a group. Resources must also be considered. The PRPs in this volunteered as part of the wider EuroTEAM project, [15] and therefore received no payment. If researchers need to recruit PRPs for specific projects, it is recommended that they follow the INVOLVE guidelines for payment [2].

PRPs in this study were involved in coding the data, and developing the themes. It was not possible to collaborate with PRPs in the earlier stages, although the patients were consulted about the search strategy and were involved in the writing of the EuroTEAM grant application. Recruiting a PPI co-applicant when writing the research proposal would allow PPI contributors to collaborate with researchers and guide the focus of the meta-synthesis. This is important as Fudge et al. [28] suggest that decisions made by professional researchers at the outset of a study have a cumulative and significant influence on the potential for PPI to impact on a study and that involvement is more difficult to achieve once studies are under way.

When reflecting on the level of control in the review process, it is important to recognise that the researchers trained the PRPs in coding so the initial phases were very much in the control of the researchers. However, as the PRPs in this study were allowed to code the data independently, they were in full control of the themes they developed. When the themes were incorporated into the researcher’s analysis, the direction, and therefore the level of control, was shared. Although the authors’ acknowledge that a PRP lead method may produce different results, this may not be as methodologically robust as PRPs are not fully trained qualitative researchers.

It is important to note that this evaluation was conducted by the researchers involved in the meta-synthesis. PRPs may have been more critical if they were asked to evaluate the study by a third party. Nevertheless, public contributors did provide some critical accounts so it seems unlikely that they struggled to articulate their own perspectives regarding training. This study also focuses on patient involvement in a qualitative meta-synthesis. Further research is needed to evaluate the involvement of PRPs in other types of systematic reviews including realist, effectiveness and quantitative meta-synthesis [21].

## Conclusion

This study adds to the existing INVOLVE guidance by informing the beginning of an evidence base on best practice regarding PPI in systematic reviews. Patient research partner insights have generated recommendations which may help researchers to involve patients more effectively in future systematic reviews. Suggestions to improve training materials for PRPs were also made. Continued monitoring and evaluation of PPI during and after systematic reviews and meta-syntheses will help researchers to optimise PPI. However, the need for on-going flexibility in each project should also be recognised in order to accommodate the unexpected and respond to opportunities and difficulties as they arise.

### Consent to publish

The authors have obtained consent to publish from the patient research partners to report individual data.

## Appendix 1: Instructions for task 1

### Patient involvement in the initial coding of data from selected qualitative papers

**Introduction**

We have gathered together papers which describe research that have interviewed patients or members of the public about their perceptions of risk and their thoughts about tests to predict future risk. We were unable to find any studies which talked about RA but we did find a few studies which talked about diabetes. We think that we could learn a lot from the studies that have taken place in other diseases such as diabetes, therefore, we’d like your help in summarising these papers.

**Patient research partner instructions**

The instructions below are a brief guide to give you an idea about what we’d like you to do. In addition to the instructions below, Rebecca will call you to talk you though each of these steps.

**The aim of this task:** We’d like to get your thoughts on the issues other studies in this area have found. We have summarised three research studies in the area of diabetes where people have describe their thoughts and beliefs about risk, and how they would feel about predictive testing. We’d like you to summarise the results section of each paper, understand the issues which are important to people who are at risk and who may be considering predictive testing:

**How to summarise:** In the document titled “activity 1 research paper results section you will find the results section of 3 papers. The results section presented in this document containing quotations from people interviewed about risk and predictive testing.

The results section also contains the thoughts of the authors, who describe concepts which they believe are important to the people interviewed. We’d like you to read through the results sections of the papers and write do your thoughts about what the researchers and people who took part in the studies are trying to communicate.

You don’t have to do all of the papers, if you feel that there’s too much work involved.

It would also help if at the end of the paper you could summarize what you feel the main issues you can see being explored in each of the papers.

We’d recommend reading the paper in small sections, and then writing a brief summary or a few words to describe the thoughts, feelings, process or behaviours being described.

When looking at each paragraph of text within the results section consider the following questions:

- What is going on?
- What is the person trying to communicate?
- What is the person saying?
- What assumptions can we make about this piece of text?

**Examples of how to summarise:** Below are a few examples of how other people have either coded text using a pen and paper, or have used a computer to do their coding:

Example 1. In this example the person summarising has chosen to print off the papers and use a pen and paper to write a few words about each paragraph. The person summarising has also highlighted important sections.

Example 2. This person has chosen to use the computer using comment boxes, to do this go to the review option in word, highlight the section of text that you want to comment on and then and select new comment.

Once you have summarised as many of the papers as you feel you want to do, please feedback your work to the research team.

## Appendix 2: Instructions for task 2 (focus group)

### Perceptions of predictive testing in those at risk of developing common chronic inflammatory conditions: a qualitative literature synthesis

**Background**

The availability of tests to predict the risk of developing chronic illness in the future is increasing. In addition, the precision and accuracy of these tests are being improved. As these tests increase in availability and precision, people who are at risk have new opportunities to understand whether they are likely to develop certain conditions in the future. The decision about whether to engage in predictive testing is often a personal one which may be driven by personal preferences for the management of risk (and risk related information), personality type and peoples personal understanding of risk and the meanings associated with being labelled as an “individual at risk”.

Previous qualitative research studies have been undertaken to explore perceptions of risk in at risk individuals, generating a broad range of important themes and topics important to consider before introducing new testing technologies. Reviews of the qualitative literature have been undertaken within the field of cancer where the area of predictive testing is seen a paramount for the early detection and treatment of disease. The themes identified within this review revealed that emotional, family and sociocultural factors can influence how patients perceive the tests. However, it is not known whether patients at risk of non-malignant disease feel the same way. Furthermore, new tests to predict the development of common chronic inflammatory diseases such as rheumatoid arthritis, and diabetes have been developed. Understanding patients’ perceptions of predictive testing for chronic inflammatory disease is paramount if new technologies are to be accepted and utilised by those at risk. However, to date, no review of the qualitative literature on patients perceptions of predictive testing for common chronic inflammatory has been undertaken.

**Aims of the review**

The aim of the review is to identify research papers which have used qualitative methods (interview or focus groups) to explore perceptions of predictive testing. The research papers included in our review focus on the perceptions of:

1. Relatives of individuals with the disease
2. Individuals with early symptoms
3. The general public

The diseases of interest for this review are common chronic inflammatory (non-malignant) diseases for which there are genetic and environmental risk factors, including the following:

1. Rheumatoid arthritis, SLE, Sjogren’s syndrome, Ankylosing spondylitis
2. Diabetes
3. Psoriasis
4. Asthma
5. Inflammatory bowel disease (Ulcerative colitis, Crohn’s disease)
6. Multiple sclerosis
7. Cardiovascular disease

**The review procedure**

Databases containing the details of published papers were searched. Because databases hold information about different papers, multiple databases were searched including MEDLINE, PSYCH-info and CINHAL. The following search terms were used across the databases above to identify relevant articles:

Perception, predictive testing, genetic, testing, risk, develop, developing, future, Rheumatoid Arthritis, SLE, Sjogren’s syndrome, Ankylosing, Spondylitis, Diabetes, Psoriasis, Asthma, Inflammatory bowel disease Ulcerative colitis, Crohn’s disease, Multiple sclerosis, Cardiovascular disease, relative, family, sibling, family history, individual, individuals, patient, people, early symptoms, symptoms, general public, people, population, healthy, qualitative.

The search strategy identified 51 potentially relevant articles. Kerin Bayliss (University of Manchester) searched through the titles and the abstracts (summaries) of the research articles to identify 10 articles at were relevant to the aims of our review.

**Data synthesis**

Kerin Bayliss initially extracted all of the relevant themes represented across the selected papers. Rebecca Stack then reviewed the papers and the extraction of themes to confirm the appropriateness of the selection of themes and the relevance of the content of the themes to the reviews primary aims.

Two (at present) patient research partners reviewed three of the selected research articles. Patient research partners coded and summarised the core themes represented in the results section of the selected articles. Kerin and Rebecca reviewed the feedback from patient research partners and absorbed the feedback and recommendations from patient research partners in to the initial thematic framework.

**Draft themes and sub themes**

1. Perceived benefits of predictive testing
  - Belief that predictive testing is reliable and effective
  - Enables the patient to gain access to an earlier diagnosis and targeted therapies
  - Potential to motivate behaviour change *(I will break this down to compare patients with high/medium/low motivation to change before the test; and behaviour change in those who receive a positive or negative test result)*
  - An opportunity to engage in altruistic behaviour
2. Barriers to the uptake of predictive testing
  - Potential for discrimination
  - Not appropriate or relevant for all conditions
  - Not appropriate for all people (*e.g., those without symptoms, without a family history of the condition*)
  - Negative perceptions of the test itself *(i.e., time consuming, need to fast. I’ll explore the different types of tests used)*
3. Lack of knowledge about predictive testing
  - Limited understanding of risk and the need for predictive testing
  - Limited understanding of what the results of the test mean
  - Conflicting views between patients and health professionals
4. Perceived impact of predictive testing
  - Predictive testing can have an emotional impact *(I will explore which patients/conditions experience a low vs high emotional impact and the reasons for this)*
  - Levels of anxiety *(I will explore which patients/conditions experience a reduction/increase in anxiety and the reasons for this)*
  - Impact of test results on family relationships
5. Recommendations for practice
  In order to learn from good practice and reduce the barriers to predictive testing to quantify risk, the literature suggests:
  - Need for a patient centred approach
  - Location should be considered
  - Need to educate patients and their families about predictive testing
  - Results should be provided by a specialist
  - Lifestyle intervention programme should be considered

**Meeting on 6** ^**th**^ **February**

In preparation for the meeting on 6th February you may find it helpful to read through the results section of the papers included in the review (attached to your email) to see if you agree with the themes and sub themes above. We will go through these themes in more detail on the 6th and there will be plenty of opportunity for discussion.

Questions you might want to consider include:

- Do these subthemes look logical?
- Based on your experience as a patient do these themes reflect reality?
- Do you think there are any important concepts described within these subthemes?
- Do you think that the overall theme or subthemes can be changed in anyway?
- Do you think that our meaning/interpretation of the quotes within the papers can be changed?
- What could be the implications of these findings for those at risk of developing Rheumatoid Arthritis in the future?
